# Fused Deposition Modelling (FDM) of Thermoplastic-Based Filaments: Process and Rheological Properties—An Overview

**DOI:** 10.3390/ma16247664

**Published:** 2023-12-15

**Authors:** Domenico Acierno, Antonella Patti

**Affiliations:** 1 Regional Center of Competence New Technologies for Productive Activities Scarl, Via Nuova Agnano 11, 80125 Naples, Italy; acierno@crdctecnologie.it; 2 Department of Civil Engineering and Architecture (DICAr), University of Catania, Viale Andrea Doria 6, 95125 Catania, Italy

**Keywords:** fused deposition modelling (FDM), thermoplastics, rheological properties, buckling, pressure-driven extrusion, flow instability, welding

## Abstract

The fused deposition modeling (FDM) process, an extrusion-based 3D printing technology, enables the manufacture of complex geometrical elements. This technology employs diverse materials, including thermoplastic polymers and composites as well as recycled resins to encourage sustainable growth. FDM is used in a variety of industrial fields, including automotive, biomedical, and textiles, as a rapid prototyping method to reduce costs and shorten production time, or to develop items with detailed designs and high precision. The main phases of this technology include the feeding of solid filament into a molten chamber, capillary flow of a non-Newtonian fluid through a nozzle, layer deposition on the support base, and layer-to-layer adhesion. The viscoelastic properties of processed materials are essential in each of the FDM steps: (i) predicting the printability of the melted material during FDM extrusion and ensuring a continuous flow across the nozzle; (ii) controlling the deposition process of the molten filament on the print bed and avoiding fast material leakage and loss of precision in the molded part; and (iii) ensuring layer adhesion in the subsequent consolidation phase. Regarding this framework, this work aimed to collect knowledge on FDM extrusion and on different types of rheological properties in order to forecast the performance of thermoplastics.

## 1. Introduction

Additive manufacturing (AM) is an emerging technology for processing material and creating an object from a three-dimensional (3D) model, usually via layer-to-layer deposition. The AM process can be constituted by two phases: the first is a virtual phase during which a computer-aided design (CAD) model is prepared using CAD software packages, the second is a physical phase to develop the physical object [1]. 

According to the standard ISO/ASTM 52900 [2], the additive manufacturing (AM) is defined: “process of joining materials to make parts from 3D model data, usually layer upon layer, as opposed to subtractive manufacturing and formative manufacturing methodologies” [3]. Historical terms are “additive fabrication, additive processes, additive techniques, additive layer manufacturing, layer manufacturing, solid freeform fabrication and freeform fabrication”.

Additive manufacturing (AM) is changing the aviation industry, space applications, and missile defense systems [4]. It opens up new opportunities for designing fine jewelry models or developing new artistic works [1], for the creation of adaptive and sophisticated buildings, or for smooth ready textures to paint surfaces [5]. The most common AM uses in biomedical applications involve the fabrication of body parts, anatomical models, implants for orthopedics, scaffolding, and drug delivery systems [6]. AM technologies are also becoming increasingly common in the automotive sector, where they are used to develop newer models and to modify existing ones in response to new design trends and technological breakthroughs [7].

The earliest patents on additive manufacturing processes were granted in 1984 to three French engineers from Cilas Alcatel (Orléans, France) (“French Patent FR2567668(B1)”) [8] and to C. Hull from 3D Systems, Inc. (Rock Hill, SC, USA) (“US Patent 4,575,330”) [9]. In the years since then, the AM sector has expanded to include a wide range of fast prototyping technologies, leading to the so-called “3rd Industrial Revolution” [10].

Controlling the rheological properties of molten/suspended polymers is critical to the efficiency of polymer processing. Controlling shear flow behavior requires the modification of many polymer properties (molecular weight, chain branching), as well as the application of modifiers (fillers, plasticizers, polymers) and adjusting processing factors (temperature, shear, pressure) [11]. 

Accordingly, the proper processing of polymeric materials requires a comprehensive understanding of their rheological properties; the characterization of polymer melt/suspended properties using relevant rheometers is very important for polymer processing management. 

This review focused mainly on research articles written in English. Scopus, ScienceDirect, and Google Scholar have been considered as three common databases. More than 1000 recent articles in the last 5 years (~50% research articles; 20% reviews; ~10% encyclopedia contributions; 20% book chapters) were identified during database searching by using the following keywords and Boolean operators: “Fused deposition modelling” OR “3D printing” OR “Fused Filament Fabrication” OR “Additive Manufacturing” AND “rheological properties” AND “thermoplastics”. Approximately 80% of the total works were eliminated: some research articles were excluded based on the title and abstract; patents, book chapters, and encyclopedia articles (often not available), theses and proceedings (except for specific cases) were not considered,. Based exclusively on the articles’ contents, 50% of those remaining (200 articles) were removed. A table of contents was developed with the following key focuses: (i) description of the fused deposition modelling (FDM) technology; (ii) the importance of rheological properties in the processing of polymer-based materials; (iii) the important role of rheological analysis in predicting material behavior in the FDM process to encourage smooth and easy operation inside the capillary extrusion while avoiding buckling and promoting dimensional stability; (iv) a summary of recent studies from the literature involving rheological testing and FDM technology; and (v) challenges and future perspectives. The review incorporated more than 100 contributions.

## 2. Fused Deposition Modelling (FDM) Technology of Thermoplastic-Based Filaments

Fused deposition modeling (FDM) is an AM technique that is commonly used to create models, prototypes, and products. FDM is a fast and simple method to make personalized items at a low cost. S. Scott Crump, cofounder of Stratasys, invented this technology in the late 1980s that became commercially available in 1990. Fast forward to today, FDM, also known as extrusion-based additive manufacturing or FFF (fused filament fabrication), is the most widely used 3D printing technology.

An STL (STereo Lithography interface format or acronyms of “Standard Triangle Language” or “Standard Tessellation Language”) file is processed by software, which mathematically slices and orients the model for the build process. Unwound from a coil, a solid filament (usually thermoplastic in nature) is heated past the glass transition/melting point and delivers material to an extrusion nozzle, which controls the flow. A worm-drive directs the filament into the nozzle at a predetermined rate. At the exit of the nozzle, the melted material is selectively deposited on a heated support platform to produce 3D parts directly from a CAD model in a layer-by-layer manner. This implies that after printing the first layer, the platform lowers and another layer is printed on top of the first one, and the procedure is repeated. The mechanism is frequently an X-Y-Z rectilinear design. A numerically controlled system can move the nozzle in both horizontal and vertical directions (along the X and Y axes), whereas the platform operates in the Z axis [12].

**Figure 1:**
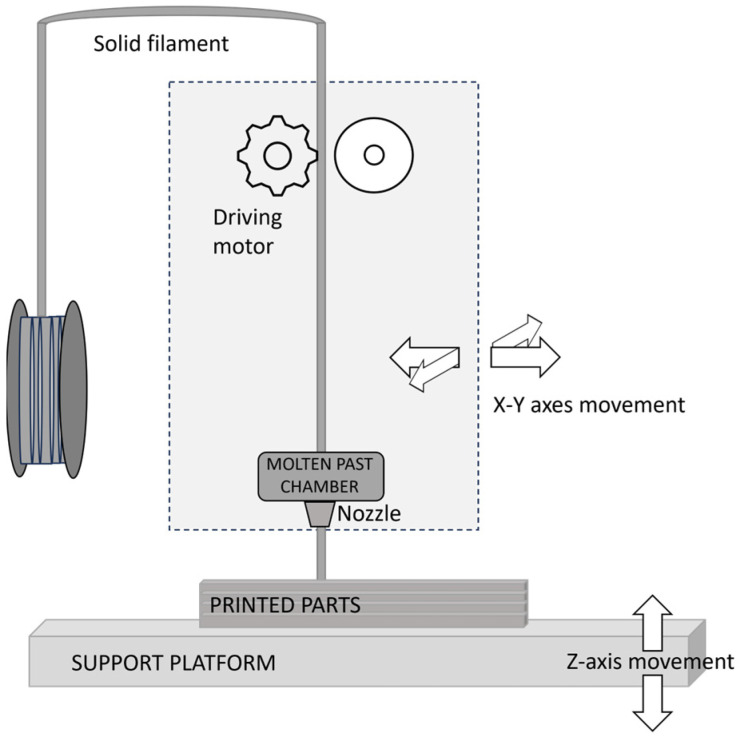
Schematic of FDM process.

A schematic diagram of the FDM extrusion process is shown in Figure 1.

Slicing parameters are layer thickness, flow rate, infill percentage, raster angle, raster pattern, air gap, nozzle diameter, and top and bottom width. Build orientation refers to the position of the part within the build platform in relation to the X, Y, and Z axes (horizontal, flat, or vertical alignment) [13]. Slicing parameters, building orientation, and temperature conditions are all considered critical parameters in the printing process [14]. 

The 3D printing process has some disadvantages, such as the low strength of the parts, rough surface finish, and slow production time [15]. For example, the layers may be too thick, leading to poor surface quality. An additional support may be needed to stabilize the 3D structure. The main cause of mechanical deficiency is deformation between layers. There is a weakness in the vertical strength of the parts due to warping and temperature fluctuations; these cause structure delamination and poor mechanical strength [14].

The maximum operating temperature of a commercially available FDM machine is commonly around 300 °C. This means that materials with exceptionally high melting points are usually inappropriate for use with this machine, and only certain types of plastics and materials that melt at moderate temperatures are suitable. ABS and PLA are the most often used thermoplastic polymers in FDM. Alternative polymers include polyamide or nylon (PA), polycarbonate (PC), polymethyl methylacrylate (PMMA), polyethylene (PE), and polypropylene (PP) [16].

Polymer-based composites and nanocomposites made with metal particles [17] (such as aluminium and iron powder), ceramics [18] (such as titanium dioxide, zirconium oxide, and aluminium oxide), nanomaterials [19] (such as carbon nanotubes, graphene, and clay), glass [20] and carbon fibres [21] (either continuous rovings or chopped fibres), and natural fibres [22] (such as wood, bamboo, flax, coir, jute, sisal, vegetable fibres, and oil palm) are commonly used in the FDM process [23].

One of the notable benefits of this growing technology is the reuse of discarded thermoplastic materials to produce quality products [24]. 

On one hand, convenient materials for extrusion-based printing are amorphous thermoplastic polymers. For amorphous polymers, internal tensions that occur after cooling (e.g., warpage) are limited by their low thermal expansion coefficients and extrusion temperatures [25]. On the other hand, semi-crystalline thermoplastics such as polylactic acid (PLA) [26] and thermoplastic polyurethanes [27] and polyamides (PA) [28] are employed to increase performance (increased toughness and wear resistance, as well as stiffness and strength) [29]. For example, semicrystalline printed parts are expected to exhibit a higher degree of anisotropy than amorphous ones. Semicrystalline polymers are also more challenging to work with when investigating interdiffusion across the contact, because incipient crystallization can be one of the limiting factors of the layer adhesion [25]. A high crystallization rate prevents interfacial adhesion, since the crystallized segment cannot weld to the prior layer; furthermore, high crystallinity causes internal stress and volume shrinkage [30].

The durability of FDM parts made from waste thermoplastics is lower than that of printed products made from virgin plastics [31]. Accordingly, it is important to understand the significant changes in recycled thermoplastic materials under repeated extrusions, such as chain fragmentation and changes in viscosity and strength at break [32]. It was discovered that the printing process has a strong impact on the pristine properties of extruded materials. This technique may induce a polymer decomposition reaction, resulting in a decrease in rheological properties over time. If the systems are kept at high temperatures, the destructive activity of the printing process can continue over time, further degrading the macromolecules. In composites, the damage caused by printing extrusion can be limited to the process duration [33]. 

Recently, new-type FDM filaments with excellent thermophysical properties have been investigated in order to improve the mechanical properties of printed parts by utilizing special engineering plastics such as polyetheretherketone (PEEK) [34], polyetherimide (PEI) [35], polyaryletherketone (PAEK) [36], and polyetherketoneketone (PEKK) [37] with excellent thermophysical properties and high melting points. This has been made possible by pushing the filament extrusion process’ technological limitations and boosting the maximum operating temperatures to 400–500 °C.

## 3. Rheology in the Processing of Polymer-Based Materials

Rheology is the science of material deformation and flow, and viscosity is an important material characteristic that indicates flow resistance. 

Consider a pair of large parallel plates, each one with area A, separated by a distance Y. One plate is set in motion with a velocity V. The space between them is filled by an incompressible Newtonian fluid. The flow is laminar. In steady-state conditions, a constant force F is required to maintain the motion of the plate. The viscosity μ is defined as constant of proportionality (for a given temperature, pressure, and compositions) between tangential force F divided by the area A (i.e., applied shear stress $\tau_{yx}$) and the velocity V divided by the distance between plates Y (i.e., velocity gradient $-\frac{dv_{x}}{dy}$):

(1) $$\frac{F}{A}=\mu \frac{V}{Y}$$

also rewritten as follows:

(2) $$\tau_{yx}=-\mu \frac{dv_{x}}{dy}$$

Equation (2), known as Newton’s law of viscosity, states that the shearing force per unit area is proportional to the negative of the velocity gradient [38]. 

This is the potential scenario of a rotational rheometer, in which a viscometric flow and a constant shear rate can be assumed across the rheometer gap. 

For incompressible generalized Newtonian fluid and for extending the principle to any arbitrary flow [39], Equation (2) is expressed as follows:

(3) $$\tau =-\eta \dot{\gamma }$$

where η can be a function of $\dot{\gamma }$(= the shear rate).

The decrease in viscosity with increasing the shear rate is referred as “shear-thinning” behaviour and the fluid is called “shear-thinning” or “pseudoplastic”. When the viscosity increases as function of the shear rate, the fluid is called “shear thickening” or “dilatant”.

Water has a viscosity of 10^−3^ Pa*s, whereas the viscosity of most polymer melts under extrusion can range from 10^2^ to 10^5^ Pa*s. Shear-rate dependency or non-Newtonian viscosity is an important characteristic of polymeric fluids during polymer processing: increasing the rate of shearing, i.e., extruding quicker through a die, reduces viscosity. This has been linked to molecular alignments and polymer chain disentanglements [40].

Various types of polymer processes often include different shear rate ranges. Compression moulding is an example of low shear rate process, whereas extrusion and injection moulding are an example of high shear rate techniques. Defining viscosity values in predetermined shear rate ranges is crucial for the design of plastic products, to ensure an easy process and to avoid clogging and loss of fluidity and productivity [41].

Polymeric fluids are referred to as viscoelastic fluids exhibiting both viscous and elastic characteristics when deformed under stress. Elastic property refers to a material’s capacity to restore its original shape after being deformed by the action of a force. Viscous property refers to a material’s irreversible deformation process, in which the basic form is no longer recoverable. The viscoelasticity of a material can be determined by applying an input and measuring the response of the material. Under linear conditions, the stress ($\tau \left(t\right))$ and the strain $(\gamma \left(t\right)$) are sinusoidal function of time (t) according to the expressions:

(4) $$\tau \left(t\right)=\tau_{0}\sin\left(\omega t+\delta \right)$$

(5) $$\gamma \left(t\right)=\gamma_{0}\sin\left(\omega t\right)$$

where $\tau_{0}$ and $\gamma_{0}$ are the stress and strain amplitude, respectively; ω is the frequency and δ is the phase angle. δ = 0° corresponds to a perfect elastic material (“Hookenian”); δ = 90° corresponds to a perfect viscous material (“Newtonian”). 

Defining the complex modulus (G*) (Equation (6)), it can be divided into two components, i.e., the storage modulus-G′ (Equation (7)), and loss modulus-G′′ (Equation (8)):

(6) $$G^{*}=\frac{\tau (t)}{\gamma (t)}$$

(7) $$G^{\prime }=\frac{\tau_{0}}{\gamma_{0}}cos\delta$$

(8) $$G^{\prime \prime }=\frac{\tau_{0}}{\gamma_{0}}sen\delta$$

G′ represents the elastic response of the material and is related to the energy stored and recovered per cycle; G″ represents the viscous response of the material and refers to the loss, or dissipated, energy per cycle. 

### Modelling of Viscosity Data

The power law (also called the Ostwald–de Waele model) is the most used model for expressing the shear-thinning behaviour of polymers:

(9) $$\eta =k{\dot{\gamma }}^{n-1}$$

where k is the consistency index and n is the power law exponent. For n = 1 the power law model reduces to Newton’s law (constant viscosity). As n decreases, the polymer becomes more shear thinning. The power law exponent (n) for the most common polymers ranges from 0.25 for poly(methyl methacrylate) and acrylonitrile butadiene styrene to 0.35 for polypropylene and polyethylene, and 0.75 for nylon and polycarbonate [42].

In addition to the power law model, two other models, i.e., the Carreau–Yasuda model (Equation (10)) and the Cross model (Equation (11)), are typically used to improve data fitting over the whole range of shear rates and to include the Newtonian plateau at low shear rates:

(10) $$\eta =\eta_{\infty }+\frac{\eta_{0}-\eta_{\infty }}{{\left[1+{\left(\lambda \dot{\gamma }\right)}^{\alpha }\right]}^{\frac{1-n}{\alpha }}}$$

(11) $$\eta =\eta_{\infty }+\frac{\eta_{0}-\eta_{\infty }}{1+{\left(\lambda \dot{\gamma }\right)}^{1-n}}$$

$\eta_{0}$ is the zero-shear rate viscosity, η∞ is an infinite-shear rate viscosity, and λ, α, and n are fitted parameters.

The zero-shear viscosity is a function of the weight average molecular weight (Mw):

(12) $$\eta_{0}=AM_{w}^{\alpha }$$

In Equation (12), the constant (A) and exponent (α) depend on polymer-based systems. Most often α is equal to 3.4. 

Viscosity dependence on temperature is expressed by Equation (13):

(13) $$\eta =\eta_{ref}\exp\left(-b(T-T_{ref})\right)$$

$\eta_{ref}$ is the reference viscosity at a reference temperature (Tref) and b is the temperature sensitivity coefficient. Most common polymers, such as polyethylene, polypropylene, polyvinylchloride, and poly(methyl methacrylate), have b values of roughly 0.01%/°C (PE), with polystyrene reaching 0.05 [42].

The temperature dependency of viscoelastic properties can also be described using the Arrhenius law (Equation (14)). For temperatures between the glass transition temperature (T_g_) and T_g_ + 100 °C, the Williams–Landel–Ferry (WLF) law (Equation (15)) was also discovered to be applicable to a broad range of polymers. The models are based on the time–temperature superposition (TTS) principle, entailing gradually translating the isotherms calculated at different temperatures through a shift factor ($a_{T}$) in relation to a curve taken at a reference temperature until all the curves overlap significantly. A master curve at the temperature of reference is obtained as a result of several shifts.

(14) $$a_{T}=\exp\left(\frac{E_{a}}{R}\left(\frac{1}{T}-\frac{1}{T_{\mathrm{ref}}}\right)\right)$$

(15) log aT⁡=−C1T−TrefC2+T−Tref

In Equation (14), Ea is the activation energy for flow, R is the gas constant. In the Equation (15), a_T_ is a shift factor, C_1_ and C_2_ are material-dependent coefficients.

## 4. Rheology in the FDM Process

The viscosity can be measured both by rotational and capillary viscometers. The ideal shear rate range during FDM extrusion is covered by a capillary rheometer, which also represents a device similar to a nozzle (i.e., Hagen–Poiseuille flow) [43]. However, during the printing process, the polymer melts undergo a wide range of shear rates, lower than 0.1 s^−1^ on the printed bed, up to 10^2^–10^4^ s^−1^ inside the nozzle [28], and a rotational rheometer can always be used. Curve fitting models (the power law, Cross, and Carreau–Yasuda models) or time–temperature superposition (TTS) can be considered useful approaches to extend the frequency scale from 0.01 to 1000 rad/s, i.e., beyond that achievable with the rotational viscometers, so as to accomplish the typical shear rate encountered by the melted polymer during the printing process [43,44]. 

However, similarities existing between the steady-state shear flow material and viscoelastic properties determined by dynamic measurements should be confirmed [28] using Equation (16):

(16) $$\left|\eta^{*}\left(\omega \right)\right|=\eta \left(\dot{\gamma }\right){|}_{\omega =\dot{\gamma }}$$

This empiricism (Equation (16)), known as the Cox–Merz rule, predicts that, for different polymers, linear and branched macromolecules, high and low molecular weights, and solutions and melts, the magnitude of the complex dynamic viscosity (η*) at frequency (ω) is comparable to the magnitude of the shear viscosity (η) at shear rate ($\dot{\gamma })$ [45].

Knowing the optimal viscosity range can help to predict whether a new melt formulation can be extruded [46,47,48]. Using a rheometer, comparing the viscosity of a new formulation with a successful extruded system is a useful method for testing the printability of a new/unknown resin. Different viscosity profiles do not always mean that the new molten formulation cannot be extruded, as long as it has a similar viscosity at the operating shear rate [43,47,49].

**Figure 2:**
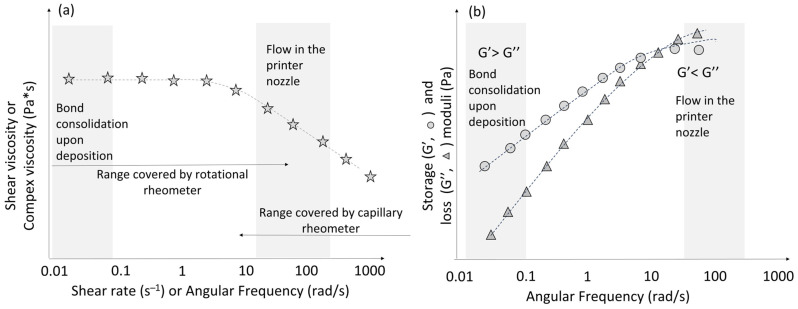
Typical viscosity (shear or complex) (**a**) and moduli (**b**) conditions for bond consolidation during deposition and flow in the printer nozzle.

Capillary viscometers are usually used for the shear rate range from about 1 s^−1^ to 10^4^ s^−1^. Rotational viscometers are usually used for the range 10^−2^ to about 10^2^ rad/s. Then, specific viscosity values should be required to extrude melted polymer through the print nozzle (shear rate in the range of 30–500 s^−1^) and during consolidation process upon deposition on the print bed (shear rate in the range of 0.01–0.1 s^−1^) (Figure 2a). The appropriate material characteristics, in terms of rheological features, are considered essential to the development of specific performance and products in the extrusion-based 3D printing process [50].

To retain the three-dimensional design after deposition, the material should have a sufficiently high zero-shear viscosity in the terminal region (low shear rate). Furthermore, a material with good printability should have shear-thinning behaviour and low melt viscosity. The shear-thinning property of the polymer promotes smooth extrusion through small nozzles in extrusion-based 3D printing [28,51]. 

Warping was found in 3D objects when the material exhibited typical polymer chain relaxation time of 2 × 10^−1^ s, in combination with elevated η_0_ values of 10^4^ Pa*s. The material’s response to thermal stress was improved, and no evidence of warpage was detected at an equal nozzle temperature (190 °C) as long as the polymer chains had a reduced relaxation time (about 10^−2^ s) and η_0_ values in the order of magnitude of 10^3^ Pa*s [44]. However, while increasing the extrusion temperature (210 °C) solved the warpage issue in the first systems (higher η_0_ and relaxation time), it decreased printing accuracy due to considerable material deburring on the edges in the second systems (lower η_0_ and relaxation time) [44].

Liquid-like characteristics (loss modulus (G″) higher than storage modulus (G′)) are also necessary to ensure extrusion through the printing nozzle. Solid-like characteristics (storage modulus (G′) higher than loss modulus (G″)) are then required to create an interlayer bond and to retain the shape in the post extrusion (Figure 2b). These conditions were validated in several investigations to demonstrate the suitability of 3D printing for various systems such as: (i) polymers (acrylonitrile butadiene styrene [52]); (ii) blends (polycaprolactone (PCL)/starch [53], poly(vinyl chloride) (PVC)/diisononyl phthalate [54]); and (iii) composites (polyamide 6/carbon fibre [28], polylactide acid (PLA)/poly(butylene adipate-co-terephthalate)/nano-talc [55], PLA/calcium peroxide [56], PLA/alumina [57], thermoplastic bio-polyurethane/carbon nanotubes [25]).

Table 1 displays the shear-thinning index and other parameters based on model fit for different polymer-based systems used in 3D printing applications.

Several critical conditions must be met for a material to be used properly in the FDM process [70]. Most of them can be summarized as follows: pressure driven extrusion at certain flow rate through an assigned diameter nozzle, ability to preserve the shape once deposited on the support throughout the characteristic processing time, dimensional stability of the extruded structure throughout cooling, and transition to the final state.

### 4.1. Buckling 

**Figure 3:**
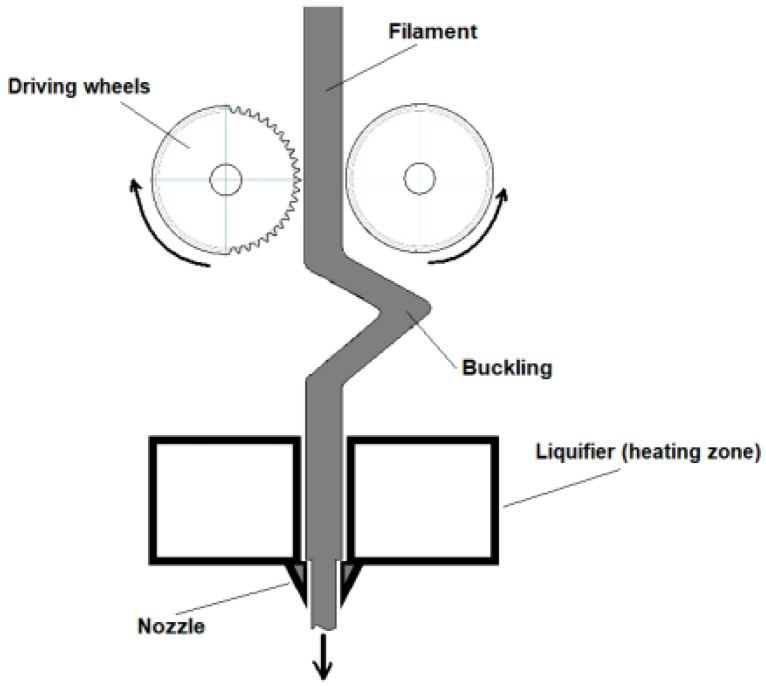
Schematic of buckling phenomena during printing. Reproduced from [72].

The printed layers must adhere well to the base support, and the printed structures must exhibit minimum warpage owing to consolidation and shrinkage. The solid filament serves as the push rod for the extrusion process. It is pulled forward by a wheel and is responsible for transmitting force to the soft material in the nozzle. Therefore, the filament must be strong enough to be handled in the printer. While it must be flexible enough to be collected as a coil, it must also be strong and rigid enough to act as a push rod during the extrusion process without breakage and deformation (buckling effect (Figure 3)) [71]. 

Two primary mechanical properties are required for the solid: enough strength to prevent buckling and enough surface hardness to prevent filament grinding induced by the extruder gears [73].

By considering L as a distance between the rollers and the printing head, a filament of radius R, K the young modulus of materials, and ${\dot{\gamma }}_{w}$ the wall shear rate, there is a critical stress limit (σ_c_) that the filament can withstand before it buckles and becomes ineffective. To avoid filament buckling, σ_c_ must be higher than the required pressure to push the filament through the nozzle (Equation (17)):

(17) $$∆P<\sigma_{c}$$

where (r and l are the radius and length of nozzle, respectively):

(18) $$∆P=\frac{2l\eta \dot{\gamma_{w}}}{r}$$

(19) $$\sigma_{c}=\frac{K\pi^{2}}{{\left(\frac{L}{R}\right)}^{2}}$$

Thus:

(20) $$\frac{2l\eta \dot{\gamma_{w}}}{r}<\frac{K\pi^{2}}{{\left(\frac{L}{R}\right)}^{2}}$$

(21) $$\frac{K}{\eta }>\frac{2l\dot{\gamma_{w}}}{r\pi^{2}}{\left(\frac{L}{R}\right)}^{2}$$

Adjusting the viscosity and shear rate could contribute to reduce the buckling [43].

Novel low-density polyethylene (LDPE) composites at 15 and 30 wt.% of waste glass were proposed to FDM for use in low-duty frictional applications. Despite having a higher viscosity during printing than the matrix, composites have a higher elastic modulus than LDPE, allowing for faster printing. The higher viscosity of composites was compensated by a higher stiffness permitting to faster filament feeding [72]. According to [49], the viscosity should not surpass specified values 10^5^ Pa*s in the shear rate period involved in printing to provide an easy flowing material and avoid buckling of the solid component of the filament.

**Figure 4:**
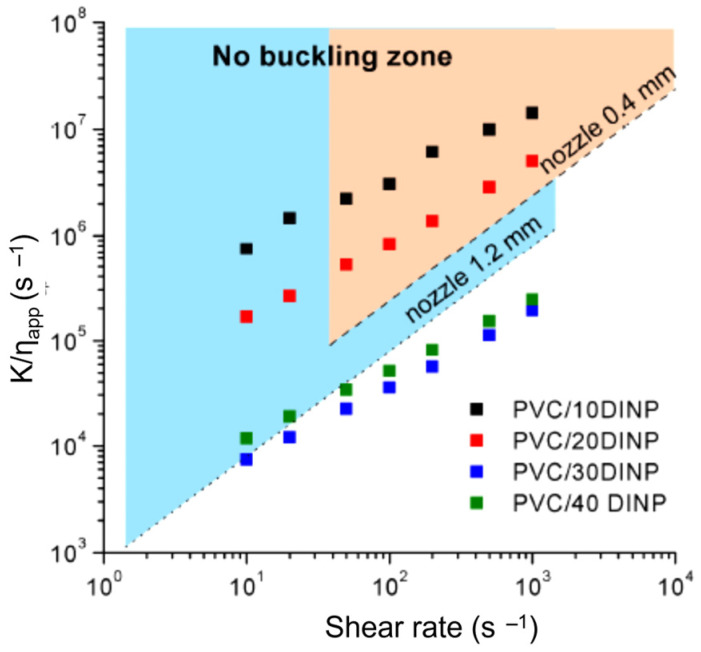
K/η_app_ vs the shear rate for formulations of plasticized PVC up to 40 wt. % of DINP content. The dotes lines highlighted critical conditions depending on nozzle diameter (0.4 and 1.2 mm). Coloured zone (blue/orange depending on nozzle diameter) indicates “no buckling” conditions. Reproduced from [54].

Rotational rheology was utilized in [54] to explore plasticized poly(vinyl chloride) (PVC) formulations, including up to 40 wt. % of diisononyl phthalate (DINP), to produce flexible and ductile filaments for use in the 3D printing process. The problem of filament buckling has been addressed by taking into account the constraint defined by the ratio of the filament’s compressibility in the solid state (K) to the viscosity in the molten state (η_app_) (Figure 4). 

### 4.2. Pressure-Driven Extrusion 

Materials with a low elasticity modulus are susceptible to buckling. As a result, if the pressure drops over the nozzle is reduced by increasing the nozzle diameter, the critical pressure for buckling increases. Thus, the processing window for a material with low elasticity modulus in 3D printer can be increased by adopting greater nozzle diameter. A variety of polymers were tested to determine the printability window [61]: thermoplastic polyurethanes (EG), ethylene–vinyl-acetates (EVA), polycaprolactone, polyethylene-oxide, hydroxypropylcellulose (HPC), polyvinylcaprolactam–polyvinyl acetate–polyethylene glycol graft copolymer, and copovidone. The filaments that buckled were discovered to be two grades of EVA and TPU with modulus values ranging from 14–70 MPa and 14–25 MPa, respectively. Optimization of process conditions, taking into account extruder temperatures (acting on viscosity) printing speed and nozzle diameter, may allow the printing of these elastic materials. Even by changing the process conditions, printing with an EVA grade (EVA2825A) failed at each nozzle diameter (0.4, 0.6 and 0.8 mm). Although EG and HPC polymers have a high elasticity modulus (442.2 MPa and 251.9 MPa, respectively), the print window expanded as nozzle diameter increased. For these polymers, the effect of nozzle diameter was correlated with another mechanism than Eulers’ buckling. This failure mechanism was linked to the processing of a highly viscous polymer. The driving force to push the melt from the nozzle in FDM is exclusively determined by the pressure drop across the system. If the pressure drop to turn the screws is excessively high due to the material’s high viscosity, the motor in the 3D printer may be unable to create the requisite torque.

The pressure driven flow of a Newtonian fluid in a capillary conduct of radius r and length l is described through Equation (22) (Hagen–Poiseuille equation):

(22) $$Q=\frac{\pi \Delta Pr^{4}}{8\eta l}$$

where Q is the volume flow rate and ΔP is the pressure drop.

For Newtonian fluids in a capillary conduct the velocity profile is parabolic, the shear rate (apparent shear rate ${\dot{\gamma }}_{app}$) at the wall is given as follows:

(23) $${\dot{\gamma }}_{app}=\frac{4Q}{\pi r^{3}}$$

A correction is necessary for non-Newtonian fluids (Rabinowitsch correction), since the velocity profile is affected by the shear-thinning behaviour of the polymer.

$\dot{\gamma }$ is the true shear rate expressed by Equation (24) whereas the pressure drop ΔP is given by Equation (25):

(24) $$\dot{\gamma }={\dot{\gamma }}_{app}\left(\frac{3n+1}{4n}\right)$$

(25) $$\Delta P=\frac{8Q\eta l}{\pi r^{4}}\left(\frac{3n+1}{4n}\right)$$

The material can be efficiently extruded if the pressure drop (ΔP) to achieve a given volume flow rate (Q) is less than the maximum pressure (${\Delta P}_{max}$) allowable by the printer apparatus:

(26) $$v_{print}\eta_{}\leq \frac{{\Delta P}^{max}R^{2}}{2l}\left(\frac{n}{3n+1}\right)$$

Given the printing machine (i.e., diameter and length of nozzle, and maximum allowable pressure drop), a specific combination of material viscosity (affected by extrusion temperature) and printing speed was discovered to cause the overcoming of ${\Delta P}_{max}$, resulting in a discontinuous flow of molten polymer from the duct and in a printing failure [44].

### 4.3. Flow Instability

Larger flow rates should be set in the typical operations of polymer processing and flow to increase productivity and reduce energy consumption. These working circumstances may be accompanied with a larger shear stress acting on the materials, causing flow instability with visible evidence of irregularity and roughness on the product surface (“sharkskin”, “stick-slip”, and “melt fracture). Causes producing extrusion instabilities are still being debated. Wall slip, melt compressibility, melt viscoelasticity, viscous heating, viscoelastic rupture, and local stick slip are all rheological characteristic of polymer-based systems that can be responsible of flow instabilities [74]. The extrudate is initially smooth and defect free at low flow rates. Defects appear on the surface as the flow rate increases (“shark-skin effect”). As the rate increases further, the flaws turn into discontinuities on the extrudate surface (“stick-slip instability”). The extrudate returns to being fault free as the flow rate is increased further in the regime known as "superflow". Gross melt fracture occurs at very high shear rates with significant irregular and chaotic surface distortions [75].

The influence of the shear rate on the shape of the PLA extrudates was shown in [76]. The extrudate had a cylindrical shape with a smooth surface at an inlet velocity of 88 mm/s (shear rate of 1560 s^−1^) and became irregular as the velocity increased up to 156 mm/s and appeared distorted at a velocity of 302 mm/s (shear rate of 5375 s^−1^).

Die swell is a typical event during the polymer extrusion due to macroscopic effects of polymer viscoelasticity such as normal stress, elastic energy, entropy enlargement, orientation effect, and memory effect. It consists of the difference in diameter between the extrudate diameter and die diameter. As a result of the action of extension, shear, and compression during polymer extrusion through the conduit, the polymer macromolecules become disentangled, uncoiled, or oriented (sheared and stretched). During die flow, the resulting stress and strain cannot be completely released. When molecules leave the die, they are loosened by elastic deformation caused by entanglement and recoiling. Extrudate tends to compress in the direction of flow and expand in the opposite direction, resulting in swelling [77].

Die swell affects final product qualities and the printing precision, and necessitates process adjustment to correct the geometric distortion [78]. According to the literature [79], swelling can be reduced by lowering the extrusion rate, raising the melt temperature, increasing the die length, decreasing the die entrance angle, increasing the draw ratio, and adding lubricants or particles to formulations. 

### 4.4. Welding

Dimensional stability of the extruded structure throughout cooling and transition to the final state was one of the investigated aspects that affected the printing quality and 3D parts properties. FDM often employs amorphous materials, which slowly change from a rubbery to a glassy state. As the material cools during this transition, the characteristics are gradually altered, and stress caused by volume variation gradually changes [80]. During the cooling stage, two neighbouring filaments should form a bond between them (intralayer bonding and interlayer bonding) throughout the viscous sintering mechanisms. 

Sintering is the phenomena of particle coalescence that is caused by two temperature-dependent properties: surface tension (driving force) and viscosity (limiting force) [81]. Particle coalescence in polymers is frequently accomplished at temperatures higher than the melting point of semi-crystalline materials or higher than the glass transition temperature of amorphous materials. The sintering process has typically been investigated for ceramics and metals, but its applicability in polymer processing has attracted the curiosity of some researchers. Traditional sintering models successfully describe polymer sintering, revealing two major elements that play a role in polymer sintering: surface tension and viscosity. Rheological properties and surface energy measurements were conducted on glycerol plasticized zeins to attest the fusion bonding behaviour in FDM process. Extrudates demonstrated a viscoelastic behaviour with a low viscosity that, for a longer time, increased due to thermal protein aggregation. The surface tension of zein-based materials was comparable to that of standard polymers used in FDM such as PLA (43 mN.m^−1^) and ABS (42 mN.m^−1^) [82].

However, further research revealed that melt elasticity is an additional influencing parameter in polymer sintering [83].

The quality of the sintering mechanism influences the bonding of the filaments once they are deposited on the heated platform, as well as the strength of the 3D parts [84]. Tensile tests were carried out on samples prepared in two different build directions. The agreement between theoretical and experimental values of the ultimate tensile load, as confirmed by microscopical examination on the fracture surface, indicated that the strength of the FDM part was mostly related to intralayer bonding, interlayer bonding, and filament neck growth [84].

At processing temperatures above the glass transition, the bond formation between melted filaments is controlled by surface contact and intermolecular diffusion of polymer chain segments across the wetted interface [85]. 

**Figure 5:**
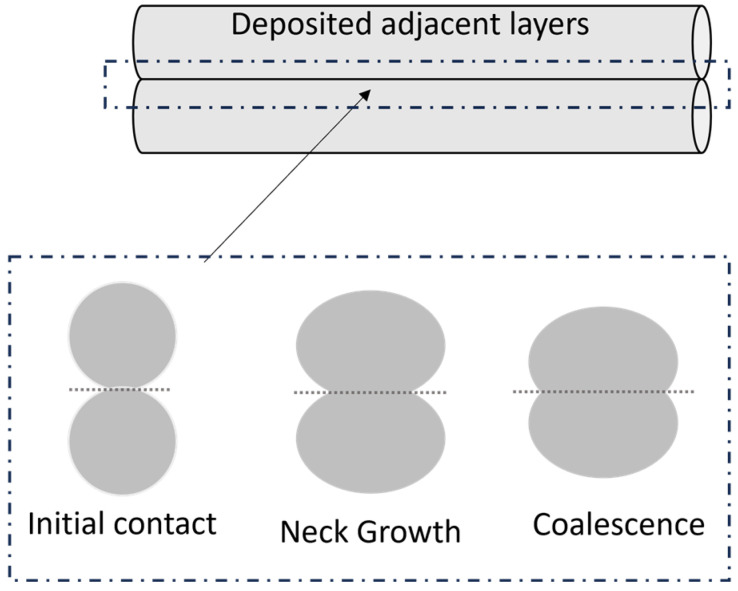
Schematic of interlayer bonding between two adjacent layers deposited on the support.

Successful bonding between two adjacent layers consists of three stages (Figure 5): surface contact, neck growth due to surface tension, and molecular diffusion and entanglement on the interface [86]. 

The successful interdiffusion and re-entanglement of the polymer melt across the layer–layer interfaces is critical to ensuring the strength of the final printed object [87]. Diffusion slows as the printed layer cools near the glass transition temperature and in response to high shear rates. High shear rates in the nozzle dramatically distort and detangle the polymer microstructure prior to welding. Because of the extrusion process’ deformation and chain alignment, macromolecular structures have a lesser capacity to migrate across the interfacial contact between two adjacent beads, to form neck growth and to coalesce [26,88]. Limits in molecular diffusion are thought to hinder interlayer contact, resulting in defects and voids. Thus, diffusion is considered to be one of the primary causes of FDM products’ poor mechanical performances [89].

Although interlayer bond strength had previously been calculated as a function of wetting process (due to surface forces) hindered by the viscous resistance, wetting was shown to be low during the rapid cooling of the deposited layer. More recently, pressure-driven intimate contact model was adopted to successfully predict interlayer contact. In other words, the bond width (W_bond_) between layers was predicted through Equation (27) [90]:

(27) $$W_{bond}=W*R_{c}{\left(\frac{P_{contact}*t_{p}}{\eta (T,\dot{\gamma })}\right)}^{1/5}$$

where $W_{bond}$: bond width; W: road width; Rc: roughness parameter; $t_{p}$: contact time of applied pressure; $P_{contact}$: contact pressure applied by the nozzle onto the freshly deposited layer (melt pressure attributed to the confined space created between the nozzle and the previous layer [91]); $\eta (T,\dot{\gamma })$: viscosity of material. It basically asserts that greater interlayer contact can be accomplished by applying higher pressures over longer periods of time to drive the layers into contact as hindered by melt viscosity.

The relaxation time, which may be calculated from rotational rheological data, is a useful material screening tool. Longer relaxation times imply that the material will diffuse slowly, which may indicate diffusive strength issues [90].

The time necessary for polymer relaxation, entanglement recovery, and diffusion to form full interfacial welding increases due to fast cooling and disentanglements. The relaxation time can be quantified by the crossover of the dynamic moduli in the low-frequency region or by dividing the zero-shear viscosity and the plateau modulus [88]. The relaxation time for high-density polyethylene (HDPE) was determined around 1–3 ms while a higher value (40 ms) was calculated for PETG. Thus, HDPE polymer chain could diffuse and weld at the interface faster than the PETG polymer chain, providing robust interlayer welding between deposited layers [88]. Both PLA and ABS have very short relaxation times (0.02 and 0.04 s, respectively). This has no substantial effect on the processing and final product characteristics because it could be assumed that the macromolecules quickly recover their equilibrium state after leaving the printing nozzle [58]. 

**Figure 6:**
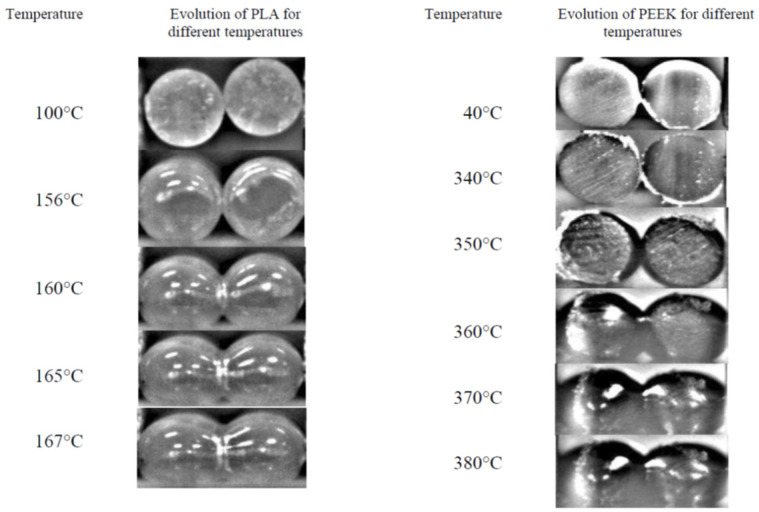
Coalescence as function of temperature for PLA and PEEK. Reprinted from [92] with the permission of AIP Publishing.

The coalescence phenomena during the FDM process of PLA and PEEK were investigated in [92]. PLA melted entirely at 160 °C and PEEK at 355 °C, and the complex viscosity of PLA in molten state was substantially lower than that of PEEK. The length of the bonding between two adjacent filaments has been registered with time and temperature (Figure 6). According to the predictive model, the experimental data demonstrated that the coalescence in PLA appeared faster than PEEK (due to the lower viscosity of PLA than PEEK). The higher the temperature, the lower the viscosity, and the stronger the coalescence. When the polymer was entirely melted, the coalescence process began.

High printing temperatures may increase the diffusion rate, but they can also promote polymer degradation [46,93], the release of more organic volatiles into the atmosphere [94], and too low molten polymer viscosity, which can induce bubble formation and flow instability [95]. Heat-induced stress is believed to cause defects and deformation in printed parts such as shrinkage and warping, which have a negative impact on printing quality [70,96]. Increasing the nozzle speed and temperatures of surrounding atmosphere resulted in less residual stress and less warpage. This is due to an improvement in heat transfer ability between the deposited layer, and to a decrease in cooling rate and thermal gradient [97].

**Figure 7:**
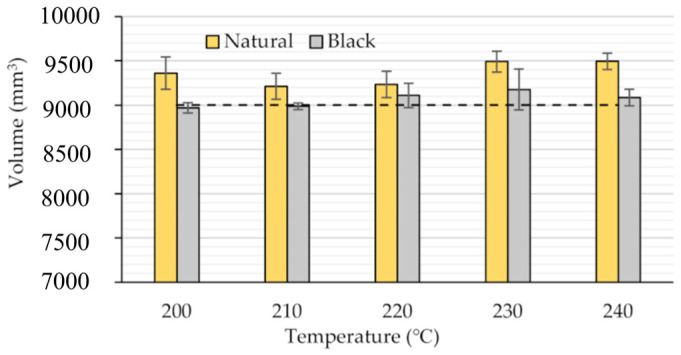
Volume of prismatic specimens as function of extruder temperature for two different types of PLA filaments (natural and black) [98].

The dimensional accuracy of the 3D printed object can be negatively affected by high printing temperatures (too low viscosities). Larger differences from the typical nominal size are represented by the higher fluidity of the polymer [98]. Using a prismatic specimen with a theoretical volume of 9000 mm^3^ dashed line, volume changes with printing temperature and filament colour (natural or black) were observed in [98] (Figure 7). The extrusion temperature has a significant impact on the dimensional accuracy of FDM-printed specimens. The dimensional errors of both kinds of PLA increased as the extrusion temperature increased. This could be explained by the higher temperature fluidity of the extruded materials, which allows the filaments to expand freely and complicates dimensional control.

**Figure 8:**
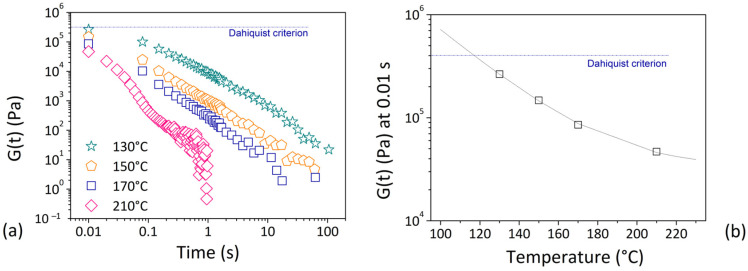
(**a**) Stress relaxation data in terms of shear stress vs time; (**b**) shear stress measured at 0.01 s as function of testing temperature. The critical Dalquist value was meet in correspondence of 120 °C. Reproduced from [99] with the permission of Chemical Engineering Transaction.

Dahlquist criterion, which was defined in the context of adhesive materials, refers to the ability to form a bond and resist debonding. Dahlquist discovered that only materials with sufficiently high compliance at the testing temperature were sticky by examining the rheological properties as a function of time and temperature. He attested the presence of a minimum value of compliance (3 × 10^−6^ Pa^−1^) or, equivalently, a maximum value of modulus (3 × 10^5^ Pa) to provide good tack or instantaneous adhesion. This criterion has been applied in the case of FDM technology to determine adhesion conditions [49]. Stress relaxation studies were carried out to determine the temperature at which the Dahlquist criterion, and hence good adhesion between layers, was satisfied. Figure 8a depicts the shear stress (G(t)) as a function of time for a recycled PLA-based filament from packaging applications. G(t) typically decreased over time, with the effect becoming more pronounced as the testing temperature rose [99]. After only 0.01 s, the shear stress measurement was lower 3 × 10^5^ Pa. This limit was met in correspondence of 120 °C by displaying G(t) value at 0.01 s as a function of testing temperature (Figure 8b). 1 sec was chosen as a typical cooling time for polymers when welding occurred [54]. Thus, once deposited on the platform, the extruded material should be at temperatures higher than 120 °C for at least 1 s to ensure good layer bonding.

## 5. Rheological Measurements to Attest the Printability of Polymer-Based Materials

Relevant rheological characterization, to attest the extrudability of materials in 3D printing technologies, consists of [50]: (i) frequency sweep test to ensure melt flow into the extruding die and potential clogging of the conducts; due to the sudden rise in viscosity and/or particles agglomeration (in the case of composite-based filaments), the arrangement of polymer chains can be hindered causing melt flow restriction and nozzle obstruction [66,100]; (ii) rheological measurements in extensional field [101] to gain information on the melt elasticity, die swell, and shape retention, (iii) transient shear stress to chain diffusion and bond healing [25,54,99], and (iv) time sweep test to verify the thermal stability of materials at the processing temperatures [102,103].

Recent research activities on new thermoplastic-based systems (blends and composites) and main results regarding the rheological properties to optimize the FDM process and meet specific requirements are detailed in Table 2.

## 6. Challenges and Future Perspectives

The challenges and future perspectives for the next generation of 3D printing technology are primarily related to increasing process and product sustainability; reducing cost and processing times, increasing the mechanical performance and durability of 3D printed parts compared to products made with traditional processes, improving the resolution and printing quality. 

Directing 3D printing towards a more sustainable development can be achieved by lowering energy consumption, using renewable natural polymers, consuming biomass sources and/ or usage of recyclable materials, reducing polluting emissions in the surrounding environment, and enabling effective waste utilization to obtain consumable products. 

Because of their low mechanical strength and thermal stability, the direct use of biomass and recycled polymers in the production of 3D printing filaments needs to be supplemented with additives, particularly macromolecules with linear structures [114]. Further study on renewable natural resources for 3D printing is needed to customize tuneable properties, such as desirable processability, printability, mechanics, bioactivity, and biodegradability [115]. New recycling procedures are required to provide recycled fibres [116] and polymers [117] with outstanding characteristics while keeping costs and carbon dioxide (CO_2_) emissions low. 

The utilization of low processing temperatures and low-emitting materials as well as establishing control measures, such as employing an enclosure surrounding the printer in conjunction with an appropriate filter, are ways to reduce polluting emissions during 3D printing [118]. 

Energy efficiency can be correlated to lower support and extruder temperatures, as well as shorter processing times [119]. This latter can be performed by increasing printing speed or reducing the melt viscosity without sacrificing the mechanical performance. 

The first point (increased printing speed) can be attained by selecting a more powerful printer and/or concentrating on machine development to help raise build speed [120]. Extrusion-based AM systems capable of producing considerably more parts than the traditional FDM technique are also currently being manufactured [121]. One example is Big Area Additive Manufacturing (BAAM). BAAM is built on a single-screw extruder. It has a large build volume, uses pelletized feedstock, and can process thermoplastics at higher rates than a filament-based system. The BAAM process allows several of the limitations of the FDM process to be overcome, such as the use of more expensive filaments in comparison to polymers in pellets form and the buckling effect during the feeding. However, this method has significant drawbacks, such as the elimination of support structures with adequate post processing, reduced printable resolution due to larger bead size, poor surface finish, and slow cooling due to quick deposition [122].

Increasing printing speed by lowering viscosity can be accomplished by using low-molecular-weight polymers and integrating flow enhancers or even plasticizers; the subsequent drop in mechanical characteristics can be addressed via reinforcement or fillers [120]. The incorporation of advanced materials as fillers into polymer matrices for FDM filaments improved mechanical properties, stiffness and toughness, thermal conductivity, electrical conductivity, and flame retardancy, broadening the range of potential applications for 3D printed components [123]. Improved thermal conductivity and lower thermal expansion coefficient, in particular, can be two critical features for promoting excellent bonding of the deposited filaments. [122]. However, one of the most significant disadvantages of filled 3D printing filaments is the potential increase in melt viscosity, which complicates the extrusion phase and raises the risk of nozzle clogging, stringing, and warping [123].

## 7. Conclusions

Among additive manufacturing technologies, fused deposition modelling (FDM) is the most widely utilized. This process consists of several stages: (i) the pushing and melting of solid filament, (ii) the material extrusion along the conduit, (iii) the deposition of melted material on a support base (at room temperature or heated) following the layer-by-layer method, and (iv) the consolidation of neighbouring deposited layers to form a three-dimensional structure. The rheological properties of molten polymers and composites used in FDM are crucial for the efficient development of the 3D printing process. A thorough understanding of the viscoelastic properties of the materials to be printed enables prediction of their behaviour throughout each step of the FDM technique. 

The efficacy of materials in 3D printing technology has been studied using both capillary and rotational rheometers. 

A good material for printing possesses a high zero-shear viscosity at low shear rates and low relaxation time and shows shear-thinning behaviour at high shear rates. The first characteristics influence the consolidation process, whilst the second is relevant to ensuring smooth extrusion through the nozzle. Given the printing apparatus, an accurate combination of viscosity (and therefore implicitly of extruder temperature) and printing speed was discovered to be determinant in favouring a continuous flow of molten material from the nozzle and avoiding a printing failure. However, too high extruder temperatures should be avoided in order to prevent polymer decomposition within the extruder chamber and poor printing quality with loss of precision in details and edges. Adjusting viscosity and shear rate could also be a valuable attempt to minimize solid filament breaking and distortion (the “Buckling” effect) when pushed and fed in the heating chamber.

Experimental evidence indicated that to favour extrusion, storage modulus (G′) should be lower than the loss modulus (G″). To maintain the shape, G′ should be higher than G″. The lower the relaxation time of materials, the higher the dimensional stability and the welding between filaments once deposited on the support plate. Finally, the Dalquist criterion was used to assess the adhesion properties between adjacent layers and the quality of layer welding. In this case, a useful condition was that the shear stress (G(t)) should be lower than Dalquist limit (3 × 10^5^ Pa).

## Figures and Tables

**Table 1:** Parameters by fitting models for various polymer-based systems used in FDM.

Materials	Shear-Thinning Index (n)	Other Parameters	Fitting Models	References
Acrylonitrile butadiene styrene (ABS)	0.27	/	Carreau–Yasuda	[58]
	0.42–0.49	k = 6678–12189 Pa*s	Power law	[59]
ABS/carbon fibers	0.50	k = 8213 Pa*s	Power law	[59]
ABS/oil palm fiber (up to 7 wt.% in content)	0.53–0.71	k = 0.0024 − 0.0008	Power law	[60]
Ethylene-vinyl acetate (EVA)	0.40–0.47	η_0_ = 5.65 × 10^2^ − 2.95 × 10^4^ Pa*s	Cross model	[61]
Polycaprolactone (PCL)	0.18	η_0_ = 4.55 × 10^3^ Pa*s	Cross model	[61]
	0.47–0.49	/	/	[62]
PCL/ hydrolyzed collagen (30 wt.%)	0.6–0.7	/	/	[62]
Polyethylene oxide (PEO)	0.47	η_0_ = 4.18 × 10^4^ Pa*s	Cross model	[61]
Polyethylene/wood flour (up to 50 wt%)	0.4	k = 10^4^–4 × 10^4^ Pa*s^n^	Power law	[63]
Poly(D,L-lactic-acid) (PDLLA)	0.33	η_∞_ = 0.1 Pa*s; η_0_ = 2260 Pa*s, λ = 0.0294 s	Carreau–Yasuda	[64]
Polylactide acid (PLA)	0.32	/	Power Law	[58]
	0.69	η_∞_ = 0 Pa*s; η_0_ = 1945 Pa*s, λ = 0.08 s	Carreau–Yasuda	[65]
	0.29–0.37	η_∞_ = 0 Pa*s; η_0_ = 1480–9460 Pa*s, λ = 0.013–0.083 s	Cross	[44]
PLA/carbon nanotubes (CNT) (1.5 to 12 wt.%)	0.51–0.07	/	Power law	[66]
PLA/ graphene nanoplates (GNP) (1.5 to 12 wt.%)	1–0.15	/	Power law	[66]
PLA/copper (66 wt.%)	0.52	k = 215 Pa*s	Power law	[67]
PLA/carbon fibers (14 wt.%)	0.76	k = 222 Pa*s	Power law	[67]
Poly(methyl methacrylate) (PMMA)/ CNT(1 wt.%)/ nano hydroxyapatite up to 10 wt.%)	0.53–0.56	/	/	[68]
Polypropylene/CNT (0.3–1 wt.%)	0.27–0.28	η_0_ = 440–509 Pa*s	Carreau–Winter	[69]

**Table 2:** Current studies from the literature on FDM thermoplastic materials, rheological characterization at specific temperatures, main results to develop specific requirements.

Thermoplastic-Based Systems	Scope	Testing	Results	Reference
Blends				
Polycaprolactone (PCL)/hydrolysed collagen (HC)	To produce biodegradable items for applications in agriculture and plant nurseries.	Capillary rheometer and flow curves at 130, 140, 150 °C	A decrease in the melt viscosity was observed with the addition of HC due to its plasticizing effect	Seggiani et al., 2018 [62]
Polybutylene succinate (PBS)/ Poly (butylene succinateran-adipate) (PBSA)	To develop semi-crystalline biodegradable filaments	Frequency sweep from 150 °C to 230 °C (TTS principle). Continuous flow measurements (Cox–Merz rule).	The viscosity values of samples lie below 10^4^ Pa*s, which allows forecasting a suitable flow in the nozzle	Candal et al., 2020 [49]
Poly(lactide) blends containing low molecular weight polymers of chemically identical but enantiomerically different nature (poly(L-lactide) (PLLA) and poly(D-Lactide) (PDLA).)	To promote interfacial weld and stiffness	Oscillatory frequency sweep tests at 180 °C	Any composition possessing η_0_ > 5000 Pa*s is too viscous for extrusion. Any composition characterized by η_0_ < 500 Pa*s, could not be printed due to uncontrolled fluctuations in volume flow rate and consequential loss in print resolution	Srinivas et al., 2020 [26]
Brominated butyl-rubber (BIIR)/polypropylene (PP) thermoplastic vulcanizate (BIIR/PP-TPV)	To flexible FDM	Frequency sweep at 180 °C	The low viscosity of blends prepared by masterbatch procedure and interfacial compatibilization effectively improves the bonding strength between the adjacent layers of the 3D printed product.	Hou et al., 2020 [104]
Polypropylene (PP)/ elastomeric ethylene-octene copolymer (EOC)	To address the deficiencies of PP in melt extrusion processing (warpage and poor layer adhesion)	Frequency sweep at 190 and 210 °C	The EOC addition did not change substantially the complex viscosity of the blends	Ho and Kontopoulou 2022 [30]
Thermoplastic starch (TPS)/polylactic acid (PLA) /poly(butyleneadipate-co-terephthalate) (PBAT) and chain extender (CE)	Highly renewable filaments for 3D printing	Frequency sweep at 180 °C.	Complex viscosity and modulus increment of blends with the addition of chain extender	Ju et al., 2022 [105]
Composites				
Wood flour (WF)/thermoplastic polyurethane (TPU) and modification with ethylene-propylene-diene-monomer grafted maleic anhydride (EPDM-g-MAH)	Adding low-cost natural fibre to make a low cost, biodegradable, and ecofriendly material	Frequency sweep at 200 °C.	Moduli and complex viscosity increase in composites after the modification with EPDM-g-MAH	Bi et al., 2018 [106]
Polylactide/hemp hurd	To valuable reinforcement of PLA-based blend	Frequency sweep at 190 °C	The biocomposites showed shear-thinning behaviour. Composites at the highest filler loading (40 wt.%) displayed lower melt flow and lower ease of processability than other biocomposite blends.	Xiao et al., 2019 [107]
Inorganic Fullerene Tungsten Sulphide (IF-WS2) nanoparticles/ poly-ether-ketone-ketone (PEEK)	Nanoparticles addition to enhance the flowability of PEEK	Dynamic shear tests at 400 °C	At low shear rate, the shear viscosity of PEEK was reduced with the addition of 2 wt% IF-WS2. The difference in viscosity of samples becomes less pronounced in the higher-shear rate range.	Golbang et al., 2020 [108]
Carbon fibre (CF)/ polyetherimide (PEI)/oligophenylene sulfone (OPSU)/polycarbonate (PC)	Plasticizing high-performance polymers	Capillary rheometer at a temperature of 380 °C	OPSU and PC decrease the melt viscosity of carbon-filled composite preserving the mechanical properties and heat resistance at a sufficiently high level.	Slonov et al., 2020 [109]
Wood flour (WF)/polyhydroxyalkanoates (PHA)	To cost reduction in PHA uses	Frequency sweep at 190 °C	The fluidity of the composites decreased with the increase in WF content	Tian et al., 2021 [110]
Polylactic acid (PLA)/ maraging steel particles	To develop composite functional filaments with additional properties (magnetic, electrical, optical)	Frequency and flow tests at 160 °C (Cox–Merz rule)	The viscous component predominates over the elastic component for all the samples. Remarkable decrease in the viscoelastic moduli as the particle content increases	Díaz-García et al., 2022 [111]
Boron nitride nanosheets/thermoplastic polyurethane (TPU)	To high-power integrated electronic devices for 5 G system	Rotational rheometer in the small-amplitude shear oscillation mode at 225 °C	G′ of the composites diverged from the behaviour of the pure TPU. However, liquid-to-solid transition not significantly affected the viscosity within the shear rate range of the 3D printing process	Gao et al., 2022 [27]
Polylactic acid (PLA)/ thermoplastic polyurethane (TPU) blend with enzymatically modified lignin (EL)	To enhance mechanical and thermal properties of bio-based polymers	Frequency sweep at 170–200 °C depending on materials	A gradual decrease in complex viscosity at higher EL concentrations	Murillo-Morales et al., 2023 [112]
Polyetherketoneketone (PEKK)/mica platelets	To provide added mechanical strength to the PEKK	Frequency sweep at 360 °C and temperature ramp	Mica doping does not significantly alter the viscoelastic properties inherent to unfilled PEKK	Kennedy et al., 2022 [37]
Poly (L-lactic acid)/cellulose nanocrystals	To produce fully green, high-performance consumables	Capillary rheology measurements and small amplitude oscillatory shear experiments	Composites exhibited shear-thinning behaviour favourable for the stable extrusion at nozzle, and G″ > G′ beneficial to the interfuse adhesion during welding	Wu et al., 2022 [113]
